# Interaction of Vanadium Complexes with Proteins: Revisiting the Reported Structures in the Protein Data Bank (PDB) since 2015

**DOI:** 10.3390/molecules28186538

**Published:** 2023-09-09

**Authors:** Marino F. A. Santos, João Costa Pessoa

**Affiliations:** 1 Associate Laboratory i4HB—Institute for Health and Bioeconomy, NOVA School of Science and Technology, Universidade NOVA de Lisboa, 2829-516 Caparica, Portugal; 2 UCIBIO—Applied Molecular Biosciences Unit, Chemistry Department, NOVA School of Science and Technology, Universidade NOVA de Lisboa, 2829-516 Caparica, Portugal; 3 Centro de Química Estrutural, Departamento de Engenharia Química, Institute of Molecular Sciences, Instituto Superior Técnico, Universidade de Lisboa, Av. Rovisco Pais, 1049-001 Lisboa, Portugal

**Keywords:** X-ray crystallography, cryogenic Electron Microscopy (cryo-EM), protein data bank (PDB), vanadium and vanadium complexes, vanadium-containing proteins

## Abstract

The structural determination and characterization of molecules, namely proteins and enzymes, is crucial to gaining a better understanding of their role in different chemical and biological processes. The continuous technical developments in the experimental and computational resources of X-ray diffraction (XRD) and, more recently, cryogenic Electron Microscopy (cryo-EM) led to an enormous growth in the number of structures deposited in the Protein Data Bank (PDB). Bioinorganic chemistry arose as a relevant discipline in biology and therapeutics, with a massive number of studies reporting the effects of metal complexes on biological systems, with vanadium complexes being one of the relevant systems addressed. In this review, we focus on the interactions of vanadium compounds (VCs) with proteins. Several types of binding are established between VCs and proteins/enzymes. Considering that the V-species that bind may differ from those initially added, the mentioned structural techniques are pivotal to clarifying the nature and variety of interactions of VCs with proteins and to proposing the mechanisms involved either in enzymatic inhibition or catalysis. As such, we provide an account of the available structural information of VCs bound to proteins obtained by both XRD and/or cryo-EM, mainly exploring the more recent structures, particularly those containing organic-based vanadium complexes.

## 1. Introduction

Many studies have addressed vanadium bioinorganic chemistry and the effects of vanadium compounds (VCs) on living beings, one of the driving forces of these research activities being their possible applications in therapeutics [1,2,3,4,5,6]. This has led to an interest in understanding interactions of VCs with proteins [1,7,8,9,10,11,12,13,14,15,16]. Namely, efforts to understand the transport of VCs in blood led to many studies of the interaction of vanadium compounds with human serum transferrin (HTF), human serum albumin (HSA), hemoglobin (Hb) and immunoglobulins [17,18,19,20,21,22,23].

For some time, studies of vanadium haloperoxidases and nitrogenases, as well as of hyper-accumulators of vanadium (e.g., some ascidians), were among the main foci of researchers, leading to the full characterization of several vanadium-containing proteins through X-ray diffraction methods (SC-XRD) [10,24,25,26,27,28,29,30,31]. The similarity between monovanadate and phosphate and studies associated with the clarification of the inhibition of ATP-dependent enzymes (ATP, adenosine triphosphate) and phosphatases by vanadate [10] also gave rise to the publication of many SC-XRD structures, including vanadate species bound to several phosphatases [10,27,28,32].

The belief that the therapeutic action of VCs may be associated with the binding of vanadium compounds to proteins has led to an increase in interest in the characterization of such interactions, namely by techniques such as single crystal X-ray diffraction analysis (SC-XRD) and cryogenic Electron Microscopy (cryo-EM). In fact, the accurate characterization of V-protein interactions is crucial to clarifying the putative role of vanadium and vanadium compounds, namely, but not exclusively, as therapeutic agents. Despite the substantial amount of information obtained using a wide range of biochemical and biophysical methods—e.g., Electron Paramagnetic Resonance (EPR), Circular Dichroism (CD), ^51^V Nuclear Magnetic Resonance (^51^V NMR), denaturing urea polyacrylamide gel electrophoresis (urea-PAGE), Small-angle X-ray Scattering (SAXS) and electrochemistry [17,33,34,35]—as recently reviewed by us [15], structural data, freely available in the Protein Data Bank (PDB), is often required to complement it [36,37,38].

PDB is nowadays a vital tool for anyone involved in protein studies from biological, biochemical or therapeutic points of view, in addition to educational purposes, as recently highlighted by the COVID-19 pandemic [39]. Following the first X-ray protein structures discovered in the 1950s and 1960s (namely myoglobin and hemoglobin by Kendrew and Perutz, respectively, who shared the 1962 Nobel Prize in Chemistry), the need for an open repository for crystallographic data rapidly arose. In 1971, the PDB was launched shortly after the Cold Spring Harbor Symposium, entitled “Structure and Function of Proteins at the Three-Dimensional Level” [40]. Since then, the number of deposited structures has exponentially increased, reaching 100,000 entries in 2014, 150,000 in 2019 and 200,000 in January 2023 (currently, in September 2023, there are more than 209,000 entries). For more detailed insights on the subject, we recommend several papers by Berman, co-founder and director emerita of the PDB, namely those published within the scope of the 50th anniversary of the PDB celebrated in 2021 [41,42,43].

The massive increase in number of available structures in the PDB is associated with the astounding technical development of structural experimental methods. In 2015 and 2021, we had the opportunity to delve into the then deposited structures of vanadium-related proteins in the PDB [10,15]. Since then, reflecting the substantial evolution of the structural biology field itself, a considerable number of new V-containing protein structures have been released. Most researchers are now more aware of the several modifications that complexes of labile metal ions may undergo when added to biological media [44,45,46,47,48,49]. In fact, the action of proteins/enzymes may be inhibited and/or modified, and the structure of the original complex may change upon binding to proteins. Thus, sorting out the changes occurring and the several types of binding that may be established between VCs and proteins/enzymes is very important, and the objective of the present review is mainly to provide an account of the presently available structural information on vanadium complexes bound to proteins. As such, in this review, we mainly propose to explore the more recent structures—obtained by both X-ray crystallography and cryo-EM—particularly those containing organic-based vanadium complexes, highlighting their contribution to a more effective knowledge on the roles and potential use of this versatile metal.

## 2. Vanadium-Containing Proteins and X-ray Crystallography

Despite the recent advent of cryo-EM, X-ray crystallography is still the top structural methodology, taking advantage of its continuous development since 1895, when Röntgen identified X-rays, and the subsequent first years of the 20th century, when its bases were postulated [50]. Such developments are recognized at different levels, namely, but not exclusively, at the level of (1) protein production and purification protocols (e.g., the use of bacterial, insect and mammal expression systems), (2) crystallization processes (e.g., automated crystallization and new crystallization additives) and (3) instrument and data-processing software improvements (e.g., brilliant synchrotron sources and fast detectors) [51,52,53,54].

The correct determination of different X-ray structures has been pivotal in many relevant chemical, biological and biochemical achievements, as attested by the numerous Nobel Prize winners in Physics, Chemistry and Medicine associated with the technique. Similarly, it is not surprising to confirm that over 85% of the total number of PDB entries have been determined using single crystal X-ray crystallography. V-containing proteins are not an exception, and most of the currently available structures in the PDB with both inorganic- and organic-based V complexes were successfully crystallized and later properly solved by X-ray crystallography. In this work, we will mainly address proteins containing bound vanadium complexes with organic molecules as ligands published since 2015.

### 2.1. Inorganic-Based Vanadium Complexes

#### 2.1.1. Monovanadates

Table 1 summarizes the released V-related crystallographic protein structures since 2015, and, with no surprise, the predominance of ortho- and metavanadate species is notorious. In fact, the similarity between monovanadate and phosphate and studies associated with the clarification of the inhibition of ATP-dependent enzymes and phosphatases by vanadate [10] also gave rise to the publication of many SC-XRD structures, including vanadate species bound to several phosphatases, namely protein tyrosine phosphatases (PTPs) [10,27,28].

**Figure 1:**
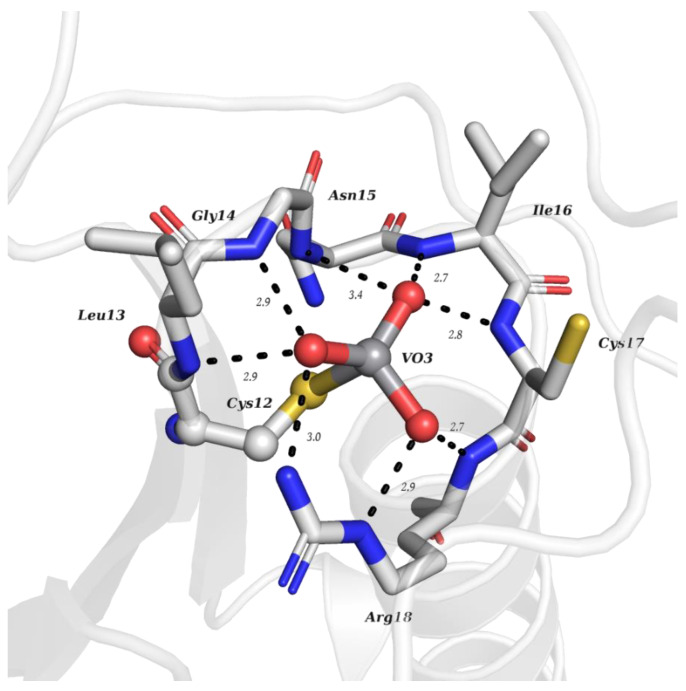
Structural representation of the VO_3_ adduct covalently bound to the Cys12 residues of bovine LMPTP (PDB: 5JNW). The axial oxygen of orthovanadate is not present, leading to a tetrahedral geometry. The adduct is also stabilized by multiple H-bonds with the residues of the so-called P-loop, a conserved active-site sequence motif, C(X)_5_R, among all PTPs (distances are represented as dashed lines and given in Å).

PTPs reverse the effect of protein tyrosine kinases by removing the phosphate group from phosphorylated tyrosine residues, controlling several cell signaling mechanisms [91,92,93]. This is the case for the low-molecular-weight protein tyrosine phosphatase (LMPTP) of the insulin receptor, putatively implicated as an anti-diabetic (type 2) biological target [94]. Stanford and collaborators solved a 1.86 Å resolution structure of the double mutated (W49Y and N50E) bovine LMPTP in the complex with vanadate and an uncompetitive inhibitor (PDB: 5JNW). Vanadate, covalently bound to the sulfur moiety of the active site, Cys12, in a tetragonal VO_3_ adduct, mimics the phosphocysteine intermediate, contributing, combined with other experimental approaches, to revealing the uncompetitive nature of the inhibitor that is nearly bound (Figure 1) [61]. As previously highlighted by Crans and co-workers [95], whether the reduction in V^V^ takes place or not depends on a combination of parameters, namely pH and oxidovanadium(V) concentration, as well as the presence and concentrations of other complexing ligands. The structure depicted in Figure 1 illustrates a V^V^–thiol binding that certainly affects the possibility of its participation in redox reactions.

Similarly, human PTP1B (PDB: 7L0H) and *Yersinia pestis* YopH (PDB: 7L0M) phosphatase variants have been co-crystallized with vanadate to investigate the role of the characteristic WPD loop in the catalytic performance [73]. Shen and collaborators proved, through kinetic studies, that both variants preserve their catalytic activity, although with a different pH activity range. Both co-crystallized 2.1 Å and 2.0 Å resolution structures support the presence of a WPD-loop closed conformation, which was later confirmed by MD (Molecular dynamic) simulations [73].

There is also an entry of a 2.15 Å resolution structure of vanadate-bound bromoperoxidase from *Corallina pilulifera* (PDB: 7QWI), which was recently released. However, the entry still lacks an associated publication.

From a different perspective, vanadate has been used as a crystallographic phasing agent. One of the main experimental challenges of X-ray crystallography is solving the so-called “phase problem” to calculate an electron density map and obtain the respective structure. Currently, due to the massive number of PDB entries, most of the new structures are solved through molecular replacement [96,97]. If no similar proteins are found, alternative methods can be used, including Single/Multiple Anomalous Dispersion (SAD/MAD) and the use of different heavy atoms (e.g., mercury, gold and platinum) for single/multiple isomorphous replacement (SIR/MIR). For a more detailed overview on those methods, including their advantages and disadvantages, we recommend specialized publications [98,99,100,101,102]. In 2020, Omari and collaborators proposed the use of vanadate to obtain experimental phases while circumventing some of the drawbacks of the traditional phasing methods (e.g., eliminating the use of selenomethionine variants). The integral membrane rabbit sarcoplasmic reticulum Ca^2+^-ATPase was incubated with orthovanadate, and a 3.13 Å resolution structure (PDB: 6YSO) was obtained from twelve different angle 360° datasets collected at a wavelength of 2.2604 Å. Despite this low resolution, the authors were able to identify two vanadium atoms in the structure (a ratio of one vanadium per 994 protein residues) combined with some other identified sulfur atoms. This strategy allowed the protein initial phases to be calculated and further improved by the subsequent steps of model building and refinement [79].

**Figure 2:**
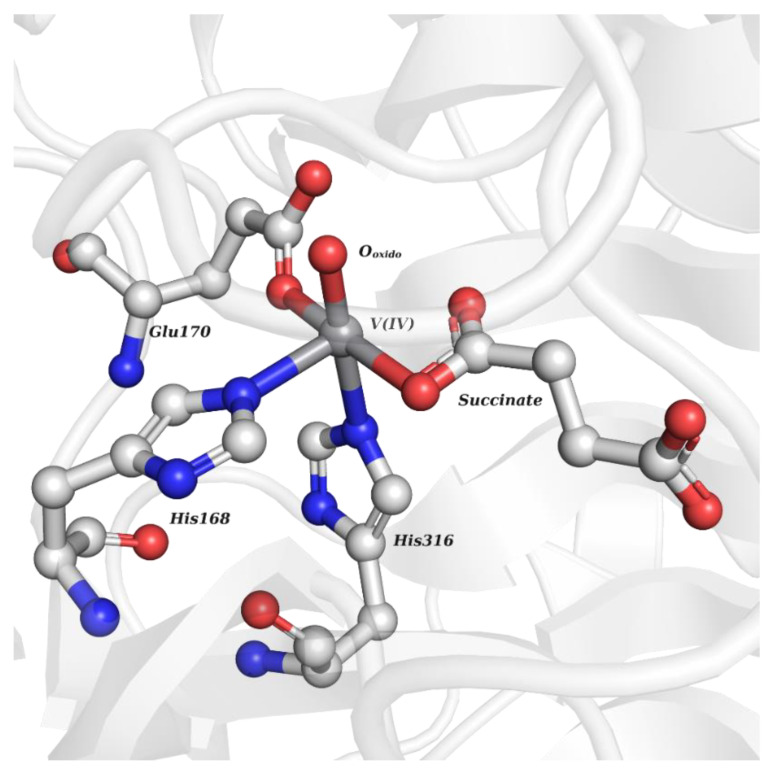
VioC-vanadyl adduct mimicking the ferryl intermediate (PDB: 6ALR). The V moiety is interacting with the side chains of three residues (His168, Glu170 and His316) as well as with one O-atom of the succinate molecule. No electron density (not represented for better clarity of the adduct) was found for the sixth coordination position of V.

Although they are less common, some protein structures (namely oxidoreductases) with oxidovanadium(IV) are also available, including two different iron(II)- and 2-(oxo)-glutarate-dependent (Fe/2OG) oxygenases known to O_2_-functionalize C-H bonds: L-arginine 3-hydroxylase (VioC) from *Streptomyces vinaceus*, which hydroxylates the C3 of the L-arginine of the antibiotic viomycin (PDB: 6ALR) [83], and the taurine 2OG dioxygenase (TauD) from *Escherichia coli* (PDB: 6EDH) [85]. Briefly, Fe/2OG oxygenases contain a Fe^2+^ cofactor that is oxidized to a ferryl intermediate (Fe^IV^-oxido), while 2-oxoglutarate is reduced to succinate. In the first case, several crystallographic VioC structures with different bound substrates and products were compared, including a 1.55 Å resolution VioC-vanadyl structure (also containing L-arginine and succinate) that mimics the unstable ferryl intermediate. The well-defined electron density of the V^IV^O^2+^ moiety exhibits a significantly distorted octahedral geometry interacting with two His and a Glu residues and with one of the O-atoms of the succinate molecule, while the sixth position is not completed (Figure 2). Further computational analysis was carried out revealing the role of Arg334 in the H-bond stabilization of the intermediate [83]. Interestingly, the V=O bond distance was determined to be 1.87 Å, which is notably longer than the theoretically established values [103], which, as reported before [104], is an artefact due to the photoreduction of vanadium during the X-ray data collection.

Finally, it should be highlighted that vanadium is also found in V-nitrogenases (V-Nases) as part of the catalytic FeVco cofactor. Nitrogenases can catalyze a reduction in atmospheric N_2_ to NH_4_^+^, thereby playing a relevant role in the nitrogen cycle. Moreover, nitrogenases can also catalyze Haber–Bosch and Fischer–Tropsch reactions under mild ambient conditions, opening up prospective industrial applications [7,105,106]. In 2021, we reviewed the most recent structural insights of V-Nases, and further information on the topic can be found in this work and the references therein [15]. Since then, a novel 1.05 Å resolution structure of the high-CO state V-Nase from *Azotobacter vinelandii* (PDB: 7AIZ) has become available. This structure presents, for the first time, a terminal CO bound to the FeVco cofactor (iron Fe6), in addition to the already characterized µ2-bridging CO (iron Fe2 and Fe6), allowing the catalytic process at the level of CO reduction and hydrocarbon formation to be seen [89].

#### 2.1.2. Polyoxidovanadates

Polyoxidovanadates (POVs), as well as polyoxidometalates in general, have been attracting increasing interest from the research community, and several prospective biological and therapeutic functions have been proposed, namely as insulin-enhancing agents [107,108,109]. Herein, we do not intend to further explore this subject, but we recommend the informative publication by Aureliano (and references therein) on the subject [110].

As expected, protein–POV interactions are crucial to their biological effects. Recent reviews explore this, including a complete structural analysis of the entries in the PDB prior 2015 (e.g., PhoE and YopH phosphatases, ecto-nucleoside triphosphate diphosphohydrolase (NTPDase) and tyrosine kinase) [15,110,111]. Protein–decavanadate ([H_n_V_10_O_28_]^−(6−n)^, V_10_) interactions are reported particularly often, but smaller POVs, including dimeric (V_2_), trimeric (V_3_) and tetrameric (V_4_) vanadates, are also reported on.

To the best of our knowledge, the currently available SC-XRD protein-V_10_ structures have all been released prior 2015. However, since then, as shown in Table 2, four additional SC-XRD structures with smaller POVs have been deposited and are briefly covered next.

Moise and coworkers were able to obtain two high-resolution (1.12 Å) variant structures (W354H and W354Y) of the PTP YopH from *Yersinia enterocolitica* with a bound V_2_ adduct (PDBs: 4ZI4 and 4ZN5). In both examples, YopH was co-crystallized with metavanadate (VO_4_^3−^) and a divanadate ester with glycerol, the latter added as a crystal cryo-protectant. The latter is observed at the active center, with one of the V moieties interacting with Cys403. The structural results, supplemented with kinetic assays using p-nitrophenyl phosphate (p-NPP) as the substrate, showed that the WPD loop is in a quasi-open position that is not compatible with a catalytically active form of YopH. As such, taking advantage of the inactive state of the protein, the adduct was not decomposed and was further characterized using computational approaches (e.g., natural bond orbital (NBO)), revealing that POVs can interact with phosphatases [58].

A 1.18 Å resolution structure of lysozyme with a cyclo-tetrametavanadate adduct (PDB: 7ZU6) was recently reported by Tito and collaborators but, so far, the associated publication is not available. A similar compound is found in other two previous structures: the ABC transporter BtuCD from *Escherichia coli* (PDB: 1L7V) and the transferase C3 exoenzyme for Clostridium botulinum (PDB 1UZI) [112,113].

Finally, Feder et al. published a 1.95 Å resolution structure of red kidney bean purple acid phosphatase bound to adenosine divanadate (see Section 2.2.1 for further details), which also contains two POV (hexa- and heptavanadate) species at the protein surface (PDB: 6HWR). Heptavanadate is known to be a partial hydrolytic product of V_10_, as reviewed by Aureliano and co-authors [111]. However, these moieties were not discussed by Feder et al., and their potential biological role was not assigned [81].

### 2.2. Organic-Based Vanadium Complexes

#### 2.2.1. Nucleotides

As previously indicated, vanadium is often used as a substrate analogue or inhibitor of a great range of enzymes. In this sense, there are quite a few examples of SC-XRD protein structures containing V moieties attached to nucleosides (replacing the phosphate group) or to nucleotides, namely adenosine diphosphate (ADP), as summarized in Table 3. All these structures contain oxidovanadates (V)-replacing phosphate, so they correspond to vanadium-substituted nucleotides.

**Figure 3:**
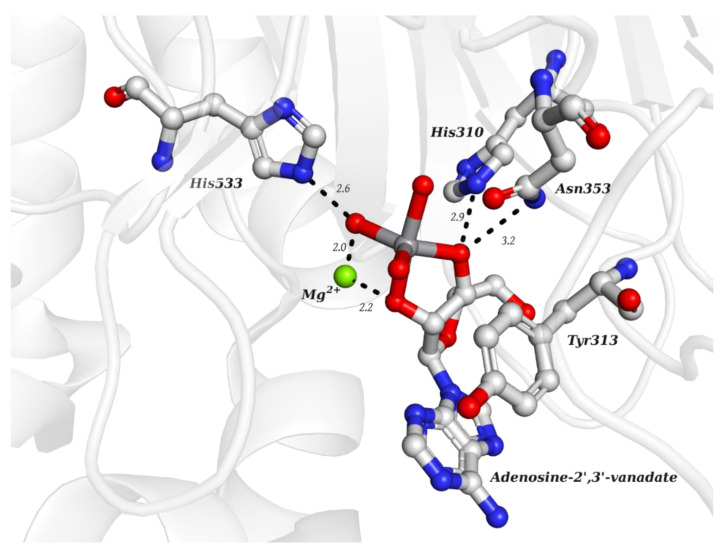
Structural representation of the adenosine-2′,3′-vanadate adduct bound to human deadenylase, ANGEL2 (PDB: 6RVZ). Different O-atoms of vanadate and ribose moieties are interacting with a Mg^2+^ ion and H-bonded to N-atoms of three protein residues: His310, Asn353 and His533 (distances are represented as dashed lines and given in Å). Tyr313 (also represented) stabilizes the adenine moiety via a π-stacking interaction.

As indicated in Table 3, there are two structures in which vanadate forms an adduct with adenosine: adenosine-2′,3′-vanadate and adenosine divanadate. In the first case, Pinto and colleagues were able to obtain a 2.10 Å resolution structure (PDB: 6RVZ) of the human deadenylase angel homolog 2 (ANGEL2) soaked with adenosine-2′,3′-vanadate, and compared it with the respective 1.45 Å resolution apo-protein structure (PDB: 6RW0), revealing, combined with a biochemical approach, its function as a 2′,3′-cyclic phosphatase [114]. During its use by the cell, RNA is known to exhibit different chemical groups at the 3′ end, namely a 2′,3′-cyclic phosphate group that is then removed by ANGEL2. Adenosine-2’,3’-vanadate, previously characterized in solution [121] and by X-ray analysis [122], mimics the transition state assumed upon 2′,3′-cyclic phosphate hydrolysis and sits at the active site next to one Mg^2+^ ion in a positively charged groove putatively involved in the binding of the RNA ribose–phosphate backbone. The adenine base establishes a π-stacking interaction with Tyr313, and the rest of the adenosine-2′,3′-vanadate moiety is stabilized by a direct coordination to Mg^2+^ and a H-network involving different (Asn353, His310 and His533) protein residues (Figure 3) [114].

In addition, as previously mentioned in Section 2.1.2, a 1.95 Å resolution structure of red kidney bean purple acid phosphatase (rkbPAP) bound to adenosine divanadate (PDB: 6HWR) is also available [81]. Purple acid phosphatases can catalyze different substrates, including ATP and ADP containing a heterovalent Fe^3+^-M^2+^ center (in which M can correspond to Fe, Zn or Mn). The apo-rkbPAP crystals were soaked with vanadate and adenosine, which formed in situ an adenosine divanadate moiety, which, in turn, is an analogue of the ADP hydrolysis to adenosine monophosphate (AMP), mimicking the expected transition state. Although the adenine base is not included in the model due to its lack of electron density considering that it is not stabilized by protein residues, docking studies supported its presence. Both divanadate and ribose moieties are visible at the active site and are stabilized by both Fe^3+^ and Zn^2+^ ions (proximal vanadate) and H-bonded to different protein residues in a five-coordinate trigonal bipyramidal geometry (proximal vanadate) and a four-coordinate tetrahedral (distal vanadate) geometry. Based on such observations, the authors proposed a rkbPAP catalytic model by which the nucleophilic O, the one that interacts with Fe^3+^ and Zn^2+^, binds to the P moiety of the substrate, allowing the opposite bond to be broken and for it to be released [81].

Uridine-2’,3’-vanadate, previously characterized in solution [121], was found in two different structures in the PDB. In the first case, uridine-2′,3′-vanadate was used as a SAD phasing agent to solve the structure of bovine pancreatic ribonuclease A (RNase), yielding a 1.90 Å resolution structure (PDB: 6YO1) (for further insights on the use of V-based compounds as crystallographic phasing agents, please consult Section 2.1.1). The data were collected at a specific V wavelength (2.2604 Å), and the protein structure was easily solved. A second data collection was carried out at a standard synchrotron wavelength (1.7711 Å), and it was still possible to determine the structure, opening up the possibility of the regular use of V-SAD phasing agents [79].

The second case is a co-crystallization 2.25 Å resolution structure of the uridine-specific Nsp15 endoribonuclease from Severe Acute Respiratory Syndrome Coronavirus 2 (SARS CoV-2, PDB: 7K1L). Kim and collaborators investigated its potential role as a therapeutic target during the recent COVID-19 pandemic, using a combined biochemical (nuclease activity assays), biophysical (differential scanning fluorimetry, DSF) and structural rationale [115]. The authors solved five SC-XRD Nsp15 structures with three different nucleotides (5′UMP, 3′UMP and 5′GpU), tipiracil (an uracil derivative synthetic substrate analogue) and uridine-2′,3′-vanadate as a transition state analogue. The last structure revealed uridine-2′,3′-vanadate moiety at the active site. The uracil base interacts with residues Ser294 and Ser346; the vanadate, covalently bound to the ribose moiety, sits where the 3′-phosphoryl group was observed (5′GpU and tipiracil structures) and is H-bonded to protein residues (His250, His235, Lys290, Thr341 and Gly247) and water molecules. Based on structural data, a two-step reaction mechanism was proposed (3′-uridine monophosphate as final product), contributing to a better understanding of the putative inhibition of the enzyme as a viable therapeutic option [115].

Both ortho- and metavanadate moieties bound to ADP molecules have been obtained, leading to a well-known model for ATP. One example is the case of a 2.25 Å resolution structure of the pre-powerstroke state of the human nonmuscle myosin-2C motor domain (PDB: 5I4E), which, using complementary mutagenesis, kinetics and molecular dynamics techniques, provided further insights into the structural changes upon F-actin binding (e.g., the existence of an allosteric communication between the distal end of the domain and the active site) [116].

More recently, the myosin-2 motor domain from the model organism *Dictyostelium discoideum* was studied by Franz and coworkers [118], who obtained two variants by replacing the two hotspot threonine residues in the connecting loop (W-loop) with two alanine residues (M765^AA^) or three glycine residues (M765^GGG^). Both M765^AA^ and M765^GGG^ variants were co-crystallized with 2 mM ADP, 2 mM metavanadate and 2 mM MgCl_2_, and two pre-powerstroke state structures were solved and deposited at a 2.60 Å resolution (PDB: 7B1A) and 2.55 Å resolution (PDB: 7B19), respectively. A structural analysis revealed a helix-mediated (W-helix) communication pathway during ATP hydrolysis, controlling the product release, while kinetics and in vitro motility assays showed that the M765^GGG^ variant significantly increased its ATPase activity, decreasing its motor ability [118].

Interestingly, the authors of the described red kidney bean purple acid phosphatase (rkbPAP) structure with adenosine divanadate (PDB: 6HWR), were also able to crystallize a 2.20 Å resolution structure of rkbPAP soaked with vanadate and ADP, leading to the in crystallo formation of ADP metavanadate (PDB: 6PY9). This substrate analogue was found at the active site and the vanadate moiety, H-interacting with both ions of the metal center (Zn^2+^ and Fe^3+^) and Asn201 through oxygen atoms and adopting a four-coordinate tetrahedral geometry that mimics the substrate-bound state. The rest of the molecule are stabilized by a H-bond network with protein residues and water molecules, even though the ribose moiety lacks them. The structure was later used in complementary docking studies with ADP and ATP, revealing additional insights into the substrate binding, which will likely be useful for future biotechnological applications [117].

Related complexes with other nucleotides—cytidine-5′-monophosphate-2′,3′-vanadate and guanosine-5′-monophosphate-2′,3′-vanadate—are also found in the PDB, interacting with ribozymes, which are well-known RNA molecules that can catalyze some biochemical reactions. We are focused on proteins, so a detailed characterization of those cases will not be provided here. Nevertheless, we note that both molecules were used as transition state analogues, enlightening the catalytic mechanisms of the respective ribozymes. Cytidine-5′-monophosphate-2′,3′-vanadate is present in two low-resolution crystallographic structures of the hammerhead ribozyme, at 2.99 Å (PDB: 5EAO) and 3.20 Å (PDB: 5EAQ) resolutions [119]. In turn, guanosine-5′-monophosphate-2′,3′-vanadate is present in two equally low-resolution structures of the pistol ribozyme, at 2.80 Å (PDB: 6UEY) and 3.10 Å (PDB: 6UF1) resolutions [120].

#### 2.2.2. Putative Therapeutic V-Complexes and Model Proteins

The use of vanadium compounds (VCs) with small organic ligands, as a means to overcome the low oral absorption rate of inorganic salts, has been common in the last few decades [123,124,125,126]. Several carrier ligands have been proposed, and an extensive number of publications cover their promising therapeutic results against different pathologies, including diabetes and several types of cancer (e.g., breast, ovarian, prostate and testicular) [1,127,128,129].

Despite the considerable amount of research on the interactions of VCs with proteins—namely blood transporters such as HTF and HSA—using a great range of experimental techniques in solution [15,17,21,130,131], the respective SC-XRD characterization has been significantly hampered. However, the use of crystallographic protein models allowed for relevant structural insights, overcoming the crystallization bottlenecks found with those blood transporters and other proteins. In the last years, some SC-XRD studies of oxidovanadium(IV and V) complexes bound to proteins have been reported, namely involving the V^IV^O^2+^ center. The first SC-XRD structure involving the oxidovanadium(IV) ion was reported in 2014 [104] and a few others have been reported since 2021 [16,75,132,133,134]. Table 4 summarizes the six VCs’ organic ligands bound to three different model proteins (lysozyme, RNase and trypsin) in the PDB, published since 2015, which will be discussed in the next sections.

##### Hen Egg White Lysozyme (HEWL)

Hen Egg White Lysozyme (HEWL) is a small anti-microbial glycoside hydrolase able to hydrolyze peptidoglycan found in bacteria’s cell walls [135]. HEWL is perhaps the most common crystallographic protein model known to produce robust and well-diffracting crystals in diverse experimental conditions (e.g., different precipitant agents and pH values). As such, there are numerous examples of soaked HEWL structures in the PDB containing different compounds, namely metal-based complexes such as those of Pt, Au, Ru, Mn and Re [136,137,138,139,140,141].

From a chronological point of view, 2014 marks the release of the first HEWL–vanadium structure: a 1.28 Å resolution structure of HEWL-V^IV^O(picolinato)_2_ (PDB: 4C3W). Briefly, we were able to model an adduct at the active site bound to the side chain of Asp52 in a distorted octahedral geometry; further EPR and DFT studies confirmed the oxidation state of the metal despite the detected long V=O_oxido_ distance due to its reduction in V^IV^ to V^III^ during the data collection [104].

Following this pioneering crystallographic structure, the first to confirm a protein–V^IV^ binding by SC-XRD, an increasing attention has been devoted to HEWL. In 2022, a thorough experimental and computational study on the binding of V^IV^O^2+^, V^IV^OL, V^IV^OL_2_ and V^V^O_2_L moieties to proteins was published, which includes the characterization of two high-resolution SC-XRDs of HEWL soaked with V^IV^OSO_4_, 2,2′-bipyridine (bipy) and 1,10-phenanthroline (phen) [16]. Complexes with both ligands were previously characterized, showing interesting anticancer and antiparasitic properties [1,46,127,142,143,144]. To further clarify their potential behavior upon protein binding, both V^IV^O-complexes with bipy and phen were used in soaking experiments with HEWL, and two V^IV^OL structures were obtained at 1.19 Å (PDB: 7Q0U) and 1.12 Å (PDB: 7Q0V) resolutions, respectively.

**Figure 4:**
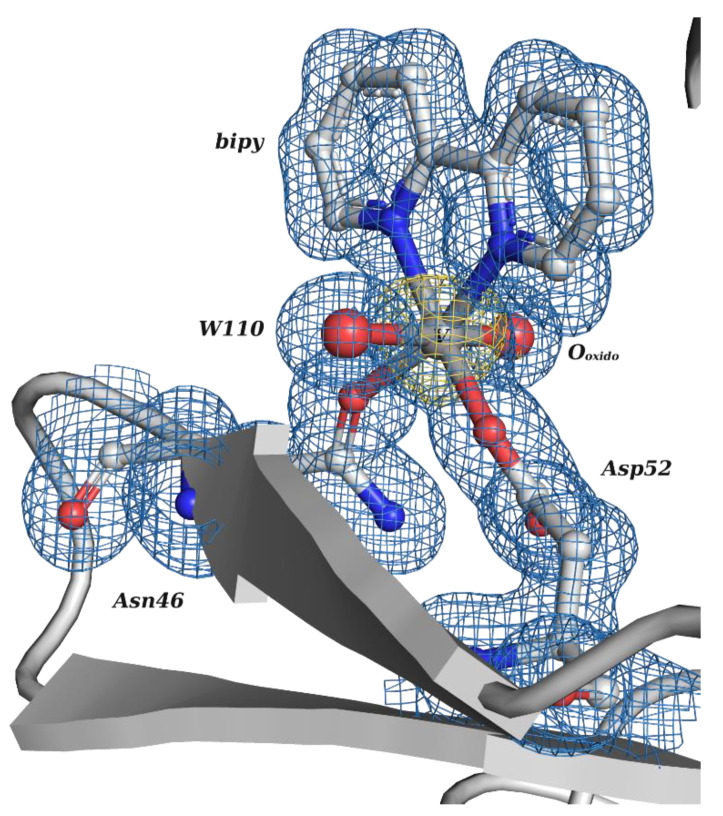
Structural representation of the V^IV^O(H_2_O)(bipy) adduct covalently bound to the Asn46 and Asp52 residues of HEWL (PDB: 7Q0U), determined by X-ray crystallography.

The first structure presents a single V^IV^O(H_2_O)(bipy) adduct at the active site (Figure 4). V adopts a nearly octahedral geometry bound to the O-donors of the side chains of Asp52 and Asn46, the two N-donors of bipy, the O_oxido_ atom and a water molecule. The adduct is also stabilized by H-bonds (Asp52 and Asn46) and by hydrophobic interactions (Glu35, Gln57 and Val109). The second structure exhibits more than one putative binding site. Similarly to the previous structure, a V^IV^O(H_2_O)(phen) adduct also sits at the active site bound to the side chains of residues Asn46 and Asp52, although with a lower occupancy and higher B factors, suggesting a more disordered moiety. It should be highlighted that additional V atoms were located next to different aspartate residues (Asp101 and Asp119), but the presence of phen moieties was not clearly revealed by the electron density [16]. Globally, this also confirms the previously indicated potential of Asp side groups to bind V^IV^O^2+^ centers [145].

The structural results described were complemented and validated by several different theoretical and experimental techniques. In brief, (1) docking simulations proved the role of microsolvation for the chiral discrimination of the binding region; (2) QM/MM experiments favored the proposed V^IV^O(H_2_O)(phen) adduct (ΔΔG_aq_ value of 5.8 kcal mol^−1^), despite the partial lack of electron density in one of the rings of phen; (3) EPR data of HEWL crystals incubated with V^IV^OSO_4_ and phen/bipy, similar to the one recorded in solution, corroborate the existence of the modeled crystallographic adducts [16].

**Figure 5:**
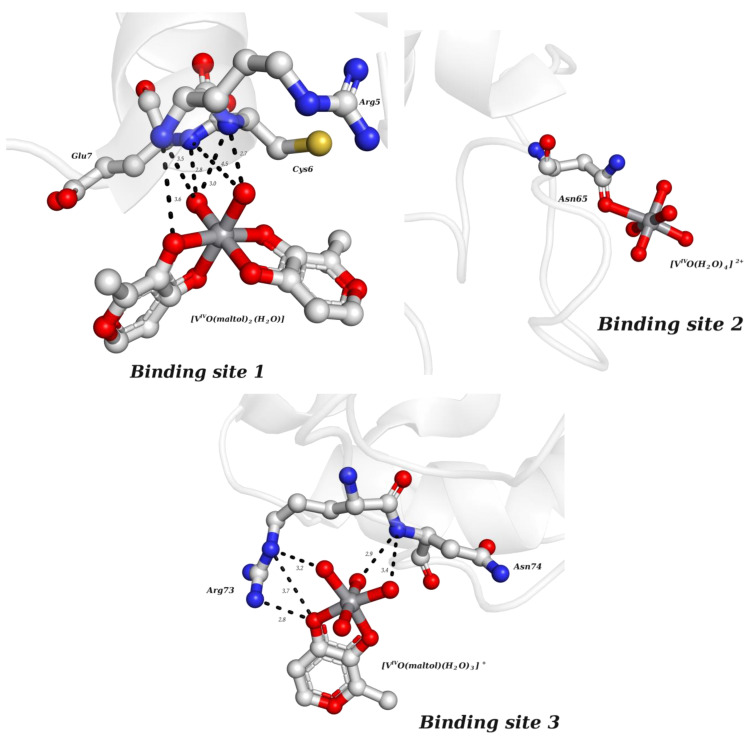
Structural representation of the adducts found in HEWL soaked with [V^IV^O(maltol)_2_] (structure A, PDB: 8AJ3). The three depicted binding sites present different V-based species, as referred to in the text, from which only the site 2 is covalently bound. H-bonds (distances are represented as dashed lines and given in Å) are important in stabilizing the adducts.

Shortly after, another work was published focusing on the ligand maltol (3-hydroxy-2-methyl-4H-pyran-4-onato) [75]. V^IV^O(maltolato)_2_ (BMOV) and V^IV^O(ethylmaltolato)_2_ (BEOV) have been among the most studied V-containing systems, including in pre-clinical tests [146,147]. Several HEWL crystals soaked with [V^IV^O(maltol)_2_] were analyzed. Two isomorphous crystals obtained from the same crystallization condition at a pH of 7.5 were tested to obtain two high-resolution structures—named A (1.13 Å resolution, PDB: 8AJ3) and A′ (1.22 Å resolution, PDB: 8AJ4)—that revealed three different binding sites (Figure 5). Three binding sites were assigned, but the nature and the occupancy of each adduct depend on the structure. Interestingly, three different moieties were found: V^IV^O^2+^, V^IV^O(maltol)^+^ and V^IV^O(maltol)_2_, arising from the original soaked compound. Structure A contains the adducts [V^IV^O(maltol)_2_(H_2_O)] (site 1), [V^IV^O(H_2_O)_4_]^2+^ (site 2) and [V^IV^O(maltol)(H_2_O)_3_]^+^ (site 3), while structure A′ presents the adducts [V^IV^O(maltol)_2_(H_2_O)] (sites 1 and 3) and [V^IV^O(maltol)_2_] (site 2) [75]. The authors discussed the binding features of each adduct, highlighting the importance of a H-network to stabilize them, which is reinforced by the covalent binding of the V moiety to Asn65 in the case of binding site 2. The EPR and ESI-MS results supported the crystallographic findings, including the +IV oxidation state. A second crystallization condition at a pH of 4 was also used for a soaking experiment with V^IV^O(maltol)_2_. However, the resulting 1.31 Å resolution structure (PDB: 8AJ5) did not present maltol moieties but three [V^IV^O(H_2_O)_3_]^2+^ ions bound to the side chains of Asp48, Asp87 and Glu35, reinforcing the labile nature of VCs upon protein binding [75].

More recently, HEWL was used to investigate the ligand 1-methyl-2-ethyl-3-hydroxy-4(1H)-pyridinone (empp) using a combined spectrometric, spectroscopic and structural approach [133]. Empp is a pyridinone derivative known for its insulin-mimetic activity by inhibiting the free fatty acid (FFA) release from isolated rat adipocytes [148]. Complex [V^IV^O(empp)_2_] was soaked into HEWL crystals obtained from distinct crystallization conditions, and three structures were determined at different resolutions: 1.08 Å (PDB: 8OM8), 1.10 Å (PDB: 8OMS) and 1.10 Å (PDB: 8OMT), designated by structures A, B and C, respectively.

Structure A, obtained at a pH of 4.0, exhibits a trinuclear oxidovanadium(V) adduct [V^V^_3_O_6_(empp)_3_(H_2_O)] as well as a [V^IV^O(empp)(H_2_O)]^+^ adduct. The first one, proving that V^IV^ can be oxidized to V^V^ under the used experimental conditions, is not covalently bound to the protein, and it is stabilized through both stacking (Trp123) and H-bond interactions (Arg73, Lys33, #Arg73 and #Asp101, where # represents a symmetry-related molecule). One of the V^V^-atoms is penta-coordinated (distorted square pyramidal geometry), while the remaining V^V^-atoms are hexa-coordinated (distorted octahedral geometry). The second one is covalently bound to the side chain of Asp48 and H-bonded to Ser50, Asn59, Arg61, #Gln121 and #Asp125. Structure B, obtained at a pH of 7.0, exhibits a single [V^IV^O(empp)_2_(H_2_O)] adduct, which is H-bonded to the main chain of Arg5, Cys6, Glu7 and #Arg14 residues. Structure C, obtained at a pH of 7.5, contains a similar [V^IV^O(empp)_2_(H_2_O)] adduct, but a second [V^IV^O(empp)_2_(H_2_O)_2_]^+^ adduct is also found to be H-bonded to the side chain of Arg125. Altogether, supported by ESI-MS and EPR data, these results emphasize the speciation of [V^IV^OL_2_] compounds upon protein interactions and the possible binding of different V-fragments, which is important to better understanding their transport in the bloodstream and possible binding to proteins inside cells [133].

##### Bovine Pancreatic Ribonuclease A (RNase A)

Bovine pancreatic ribonuclease A (RNase A) is one of the most representative ribonucleases and has been extensively discussed in the literature over the years [149,150,151]. Its relevance is reflected by the attribution of the Chemistry Nobel Prize in 1972 (Anfinsen, Moore and Stein) and 1984 (Merrifield) to research involving this protein.

RNase A is widely used as a model protein, including in metalation- and crystallization-related investigations [152,153,154]. To the best of our knowledge, there are currently two SC-XRD structures of RNase A soaked with an organic-based vanadium compound. The first one is a 1.27 Å resolution structure (PDB: 7P8R) [132]. This structure exhibits two molecules (A and B) in the asymmetric unit, but only one adduct is bound to the side chain of the Glu111 residue of molecule A. V^IV^ presents a slightly distorted octahedral geometry with a bidentate coordination of the two picolinato ligands, an O_oxido_ atom and an O atom from the referred Glu111 and is further stabilized by H-bonds. The bond lengths within the adduct present values in the expected range, even if the V^IV^=O_oxido_ (1.68 Å) is marginally larger than the theoretical values, as found in the concomitant HEWL-V^IV^O(picolinato)_2_ structure [132]. Globally, this also confirms the previously indicated potential of Glu side groups to bind V^IV^O^2+^ centers [155]. The authors used different experimental methods—namely ESI-MS, CD and EPR—to consubstantiate these structural results. Briefly, ESI-MS proved that the adduct RNase A-[V^IV^O(pic)_2_]−[phosphate/sulfate] is formed at 14,088.5 Da; CD showed that the secondary structure of the protein is not altered upon ligand binding and EPR suggested that the equatorial water molecule of the original compound [V^IV^O(pic)_2_(H_2_O)] is replaced by the protein residues Asp/Glu or His, depending on the pH (acidic or neutral, respectively). Further computational approaches confirmed its binding to Glu111 at acidic pH values while also predicting its binding to His105 and/or His119 at physiological pH values. Interestingly, the authors also explored the potential protein inhibition caused by [V^IV^O(pic)_2_(H_2_O)], verifying that the catalytic activity of RNase A is significantly reduced in its presence. This finding agrees with the structural data, as the adduct sits near the active site of the protein [132].

The second is a 1.57 Å resolution structure (PDB: 7QWH), obtained in a recently published relevant work from Ferraro et al., involving UV-vis, circular dichroism, EPR, computational and X-ray crystallographic studies on the system bovine RNase A with [V^IV^O(8HQ)_2_] (8HQ = 8-hydroxyquinolinato) [134]. From the XRD analysis, it was found that the geometry around the vanadium center corresponds to a slightly distorted square pyramid; one of the 8HQ ligands is replaced by a water ligand of the OE1 of Glu111, yielding a [V^IV^O(8HQ)(H_2_O)]^+^-RNase A adduct. The 8HQ is maintained in its position through stacking interactions with the His119 side chain. The O-atoms within the vanadium coordination sphere are H-bonded to the N-atoms of the side chains of Asn71 and Gln69 and to H_2_O molecules. The V^IV^ = O_oxido_ distance is 1.66 Å, thus within the expected range for these bonds. DFT and docking calculations allowed for a deeper understanding of the system; namely, from the crystallographic data at a pH of 5.1, it was concluded that the formation of the [V^I^O(8HQ)(H_2_O)] + -RNase A adduct explains/results in the inhibition of the RNase A activity [134].

##### Bovine Trypsin

Serine proteases, which are able to hydrolyze peptide bonds within proteins, are an important family of enzymes that are involved in multiple biological processes and signal transduction pathways, including digestion, immune response, blood coagulation and apoptosis [156,157,158,159]. Bovine pancreatic trypsin is one of the most typical serine proteases and is commonly used as a model in related studies. From a crystallographic point of view, trypsin is also an interesting model as it produces well-diffracting crystals under a significant range of experimental conditions.

There are currently two X-ray bovine trypsin structures deposited in the PDB containing vanadium compounds (picolinato and 1,10-phenanthroline), released in 2022 [16]. Before 2022, only a 1.5 Å resolution structure of the related bovine chymotrypsin with vanadate and benzohydroxamic acid (PDB: 2P8O) was available; this showed an adduct at the active site with a distorted octahedral geometry and the V atom covalently bound to the O atom of Ser195 [160]. We highlight that the binding to the side group of serine confirms the findings of earlier studies indicating its potential to bind V^IV^-centers [161].

Focusing on the available V-trypsin structures, a co-crystallization strategy with V^IV^OSO_4_ + picolinato was followed, and a 1.09 Å resolution structure was obtained (PDB: 7Q0X). A single V^IV^O(picolinato)_2_ adduct, very similar to the one described with HEWL, sits at the active site, with V^IV^ presenting a distorted octahedral geometry bound to Ser195, an O_oxido_ and two picolinato anions through the N and O atoms. The adduct is further stabilized by hydrogen bonds (Ser195 and Gln193) and hydrophobic interactions (Phe41, Cys42, Cys191, Gln192 and Phe215). Despite its resemblance with the HEWL adduct, the V^IV^ = O_oxido_ bond length is significantly shorter (1.70 Å versus 1.82 Å); being slightly above the usual values (1.57 to 1.65 Å) [71], the result was interpreted as being due to a partial reduction of the metal during the data collection. Additional docking studies corroborate the structural results when both buried and surface water molecules were included, proving, as before for HEWL, the essential role of microsolvation for the chiral discrimination of the binding region [16].

**Figure 6:**
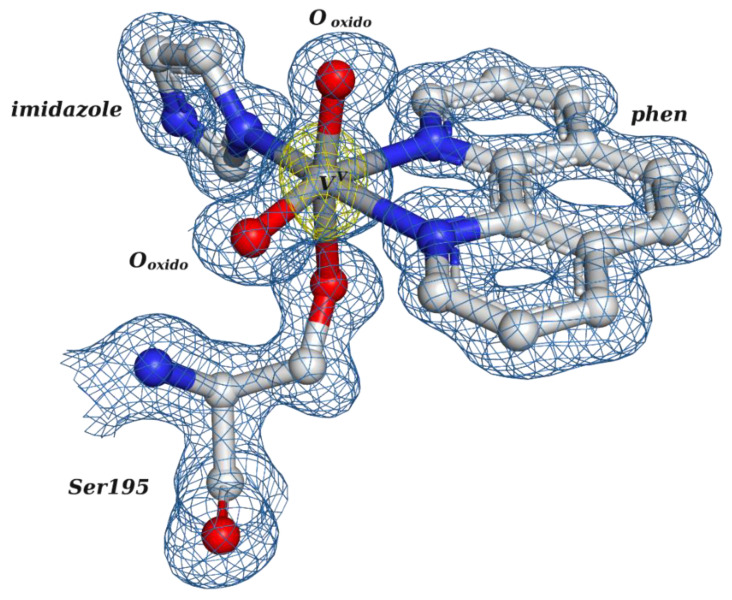
Structural representation of the V^V^O_2_(phen)(imidazole) adduct covalently bound to the Ser195 residue of bovine trypsin (PDB: 7Q0W), determined by X-ray crystallography.

The second V-trypsin structure was motivated by the fact that the previously referred-to HEWL-V^IV^O(H_2_O)(phen) adduct is partially lacking in electron density for the phen moiety. A successful co-crystallization experiment with bovine trypsin resulted in a 1.20 Å resolution structure (PDB: 7Q0W). The structure revealed multiple imidazole (Im) molecules arising from the crystallization condition and a single V adduct at the active site with a distorted octahedral geometry (Figure 6). V is bound to the O-atom of the side chain of the Ser195 residue, to the two N-atoms of the phen moiety, to the N-atom of an imidazole molecule and to two O-atoms. Similarly to picolinato, hydrogen and hydrophobic interactions have a relevant role in stabilizing the adduct [16].

The distances between the V moiety and those O-atoms (1.70 and 1.71 Å) did not reveal the adduct as V^V^O_2_ or V^IV^O(OH). Computational methods—DFT and docking simulations—were used to address this point, showing that the presence of two O_oxido_ or a (O_oxido_, OH) couple is equally possible (similar energies, although the V^V^ moiety is slightly preferred) [16]. The finding that the conversion energy between the two common vanadium oxidation states (IV and V) is low suggests that the interchange of the two forms of the VO(phen)-trypsin complex is easy, anticipating consequences for the ease of ROS production. It is not yet known whether this will happen for other complexes and proteins, but this is a subject to be properly explored by researchers in the near future.

## 3. Vanadium-Containing Proteins and Cryo-EM

Despite the still vast prevalence of structures determined by X-ray diffraction techniques when compared with those determined by other methods, structural biology is witnessing a paradigm shift: seven years ago, 89% of the total number of PDB entries were solved by X-ray crystallography, while, in 2023, this percentage decreased to 85%.

**Figure 7:**
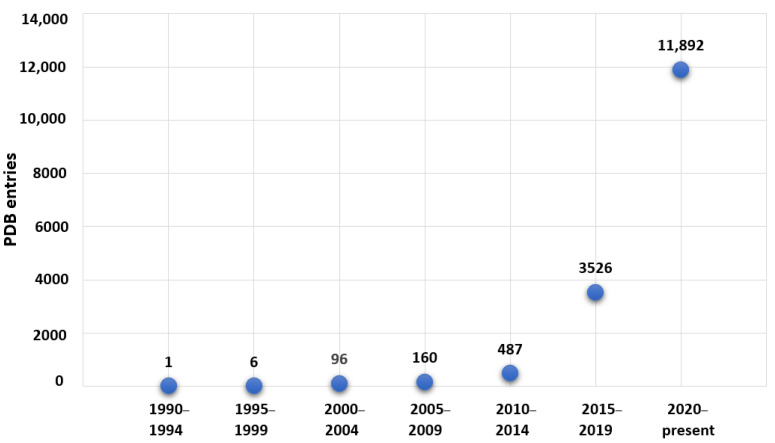
Evolution of the cryo-EM structures deposited in the PDB up to July 2023 over 4-year periods.

One of the main reasons for this scenario is the rise of cryo-EM techniques—in particular, single-particle analysis and cryo-electron tomography—which were further recognized by the attribution of the Chemistry Nobel Prize in 2017 to Dubochet, Frank and Henderson. In the recent years, cryo-EM has made remarkable technical progresses and much higher resolutions are now possible. Simultaneously, access to cutting-edge equipment is now facilitated and diffused throughout the worldwide community dedicated to structural resolution. Altogether, considering its potential to better mimic physiological conditions as it may be applied to samples in solution, cryo-EM has become one of the most popular structural methodologies. In fact, as expressed in Figure 7, there is an exponential increase in the number of PDB entries solved by cryo-EM methods, and approximately 74% of them have been deposited since 2020. For a more detailed description on the advances and potential uses of cryo-EM (including in drug design projects), we recommend several publications [162,163,164,165,166].

Table 5 summarizes the vanadium-containing protein structures that have been deposited in the PDB since 2015. These show a clear incidence of ADP-vanadate species that are used to mimic the intermediate state of the ATP hydrolysis of several ATP-binding cassette (ABC) transporters.

It should be noted that many of the structures identified as containing inorganic orthovanadate (identifier VO4) also contain an associated ADP moiety, as the authors did not deposit the ADP-vanadate as a single molecule (identifiers AOV or AD9). In fact, from the eleven VO4 structures indicated in Table 5, only one contains exclusively orthovanadate; it is a 3.30 Å resolution structure of an *E. coli* potassium uptake transporter KdpFABC (PDB: 7ZRD). KdpFABC contains different subunits, namely the P-type ATPase KdpB, in which a serine residue (Ser162) is phosphorylated when no more potassium is needed, leading to the inhibition of the transporter. To further elucidate this inhibition from a structural point of view, Silberberg and collaborators characterized a vast range of cryo-EM wild-type and variant KdpFABC structures resulting from inhibiting and non-inhibiting conditions, and a new inhibited KdpFABC state (named E1P tight) was found [174].

Taking advantage on its well-known structural similarity with phosphate, orthovanadate (able to stabilize P-type ATPases in an E2P state) was incubated with a wild-type KdpFABC sample (PDB: 7ZRD). The orthovanadate was found next to the residue Asp307 of KdpB, known to be bound to the γ-phosphate of ATP. Interestingly, the cryo-EM structure was determined in an E1P-tight state similar to the one under turnover conditions (PDB: 7ZRE), which was interpreted as the conformation adopted after ADP release [174].

Regarding ADP-vanadate intermediate conformation form structures, we recently listed some of them [15]. Our review encompassed the single particle cryo-EM structures of the following ABC transporters: (1) LptB2FG and the complex LptB2FG-LptC from *Escherichia coli* (PDBs: 6MHZ and 6MI8) [176]; (2) TmrAB from *Thermus thermophilus* in two different conformations (PDBs: 6RAK and 6RAJ) [177]; (3) NaAtm1 from *Novosphingobium aromaticivorans* (PDB: 6VQT) [167]; and (4) MlaFEDB from *Acinetobacter baumannii* (PDB: 7D0A) [169].

Since then, a considerable amount of other bacterial ABC transporters cryo-EM structures containing an ADP-orthovanadate adduct have been made available. This is the case for a 3.50 Å resolution structure of the *E. coli* ABC transporter complex LolCDE (PDB: 7MDY), which is able to transport lipoproteins from the inner membrane to the outer membrane. The authors were able to obtain an intermediate conformation of the ATPase with one ADP-vanadate moiety in each of the two ATP sites at the interface of a dimer of the LolD domain. This binding led to the rearrangement of the transmembrane helices (TM2) in the domains LolC and LolE which, in turn, released the bound lipoprotein to the periplasm [178].

There is also an example of a cryo-EM entry containing an ADP-metavanadate adduct, the 3.90 Å resolution structure of the ABC transporter MlaFEBD from *Pseudomonas areuginosa* (PDB: 7CH8), which participates in the transport of phospholipids to the inner leaflet of the outer membrane in Gram-negative bacteria. To gain further insights into the transport mechanism, the authors determined and compared three distinct cryo-EM MlaFEBD structures: the apo-form (nucleotide-free), the previously referred-to ADP-vanadate (mimicking the ADP-bound post-hydrolysis conformation) and the ATP analogue AMPPNP (mimicking the ATP-bound state). Unexpectedly, significant conformational modifications upon nucleotide binding were not detected. An ADP-vanadate moiety was found at the ATP binding site of each copy of the MlaF dimer. The adenine and the ribose moieties are coordinated to Arg18 and Arg21, respectively, while the phosphate moieties are coordinated to Lys47 and a magnesium ion, and the vanadate is close to the catalytic Glu170 residue. Several phospholipids were found to bind to the substrate-binding pocket of the MlaE, suggesting that this step can be a passive diffusion mechanism rather than an ATP-dependent one [181].

Much less commonly, an eucaryotic ABC transporter with ADP-orthovanadate is also found in the PDB: a single particle 3.77 Å resolution structure of Pdr5 from *Saccharomyces cerevisiae* (PDB: 7P06). Pdr5 is an efflux pump belonging to the PDR (pleiotropic drug resistance) subfamily, which is deeply involved in multi-drug resistance as it can transport a great range of xenobiotics. Known as a model for pathogen fungi (e.g., *Candida albicans*) homologues, different apo- and nucleotide-bound Pdr5 were characterized. An ADP-orthovanadate structure was found in an outward-facing conformation, corresponding to intermediate step of the ATP hydrolysis. The authors further explored the structural rearrangements in both transmembrane and extracellular domains, revealing an asymmetric ATP hydrolysis as conformational changes are much more pronounced in one half of Pdr5, supporting a peristaltic movement of the xenobiotic [179].

Finally, cryo-EM was also used to characterize a system containing a polyoxidovanadate. Their putative biological and therapeutic relevance were mentioned in Section 2.1.2 [107,108,109,110,111]. Among polyoxidovanadates, decavanadate (V_10_) has been particularly studied [182]. Winkler and collaborators were able to solve a 3.80 Å resolution single-particle structure of the human ion channel TRPM4 complexed with V_10_ (PDB: 5WP6) [175]. TRPM4, belonging to the TRPM (Transient Receptor Potential Melastatin) subfamily, is a Ca^2+^-activated non-selective channel that transports sodium and/or potassium to depolarize the cell when the intracellular calcium level increases, participating in countless bioprocesses such as the cardiac rhythm. The study aimed to characterize the V_10_ binding to the protein as V_10_, being highly negatively charged, interferes with the membrane potential, modulating the voltage dependence of TRPM4. Two V_10_ binding sites were found (exhibiting several positive residues), at the turn of the C-terminal domain and at the interface of MHR1/2 and the MHR3 of the MHR (N-terminal TRPM homology region) domain. The different structural aspects were discussed (e.g., the role of Gln977 in the Ca^2+^ permeability of the protein), contributing to elucidating the function of the TRPM ion channels [175].

## 4. Conclusions and Future Directions

As widely described, X-ray crystallography and, more recently, cryo-EM have played pivotal roles in shedding light on the current state of the art of several biological processes. Despite the massive advances in the last decades, much more is expected and will certainly be achieved in the next few years, namely by the introduction of artificial intelligence (AI) tools, currently mainly represented by AlphaFold software (https://alphafold.ebi.ac.uk/, accessed on 7 September 2023) [183].

In fact, the technical development of both experimental and computational structural methods and the understanding that the biological effects of metal complexes on biological systems, namely of vanadium complexes, may be associated with interactions of the metal compounds with proteins, led to an enormous growth in the number of reported structures in the PDB. Concomitantly, this led to a much better understanding of the nature and variety of interactions of metal complexes with proteins, as well as the mechanisms of many reactions involved either in inhibition or catalysis of enzymes by metal ions or in many other types of involvement of metal complexes (e.g., structural or toxic effects) that impact on proteins.

Regarding vanadium compounds, besides halogenases, nitrogenases and vanabins, for which structural information has been available for some time [1,7,10,24,25,26,27,28,29,30,31], and the interference of vanadate in several biological processes [6,7,9,184], it is presently recognized that the biological effects of VCs may be due to their interaction with proteins, and several studies have addressed this topic. The number of structural studies addressing the interaction of vanadium compounds with proteins will undoubtedly increase in the near future and will provide new information and clues to help understand the biological and therapeutic effects of VCs.

Notwithstanding, despite the relevance of information provided by the several methodologies used, it is known that in biological media, VCs undergo hydrolysis and will certainly be involved in several ligand exchange and redox processes, as well as modifications in geometry, coordination number and nuclearity [15,111]. Additionally, at the low concentrations prevailing, the species formed may totally differ from the compound initially introduced in the media; thus, if a biological effect is reported, the mechanism of action proposed must take into account the species that are effectively formed. Most researchers are now more aware of the complex speciation that is established in biological media [44,45,46,47,48,49]. Thus, the understanding of the changes occurring and of the several types of binding that may be established between VCs and proteins/enzymes is very important, not only because their action may be inhibited and/or modified but also because the structure of the original complex may have changed upon binding to the protein. The present review provides an updated account of the presently available structural information of vanadium complexes bound to proteins, and it was indeed confirmed that in many cases, the V-containing species that is found to be bound to the protein differs from the one initially added to the crystallization media.

## Figures and Tables

**Table 1:** SC-XRD structures of monovanadate-containing proteins available in the PDB since 2015. The identifier and the name/chemical structure of each V compound, as well as the respective PDB codes, are provided. ^a^ The publication associated with the PDB entry is not available.

V Species Identifier	V Species Name/Chemical Structure	PDB Codes
V	Vanadium ion	6DYH [55], 6DYL [55], 7Q0T [16]
VO4	Orthovanadate V^V^O_4_^3−^ 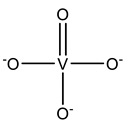	4RI4 [56], 4ZG4 [57], 4ZI4 [58], 4ZN5 [58], 5AA6 ^a^, 5BZX [59], 5HMP [60], 5I0I [60], 5JNW [61], 5N69 [62], 5OFR [63], 6DR7 [64], 6DT6 [64], 6I7E [65], 6LVQ [66], 6NPZ [67], 6PG0 [68], 6PGT [68], 6PHA [68], 6PHS [68], 6PM8 [68], 6S3N [69], 6XEA [70], 6XEF [70], 6YCX [71], 6YSY [72], 7EZN ^a^, 7L0H [73], 7L0M [73], 7MPC [74], 7QWI ^a^, 8AJ5 [75]
6BR	Threonine-vanadate V^V^C_4_H_8_NO_7_ 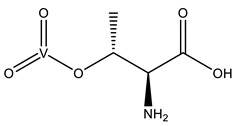	5IJS [76]
VN4	Metavanadate V^V^O_3_^−^ 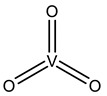	4RI5 [56], 5A3Q [77], 5A3S [77], 5DMP [78], 6YSO [79]
VN3	Metavanadate V^V^O_3_^−^ 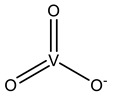	5Z5A [80], 6HWR [81]
VVO	Oxidovanadium(2+) V^IV^O^2+^ 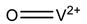	5BK9 [82], 6ALR [83], 6DB2 [84], 6EDH [85], 6VWQ ^a^, 6VWR ^a^, 6XPA [86], 6ZAK ^a^, 8AJ5 [75]
VVB	Bis(oxidanyl)vanadium V^II^(OH)(OH) 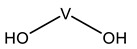	8AJ5 [75]
H1W	Pentakis(oxidanyl)vanadium V^V^H_5_O_5_ 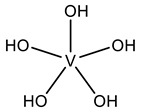	6HWR [81], 8AJ3 [75]
D6N	FeV-Center VCFe_7_NS_7_ 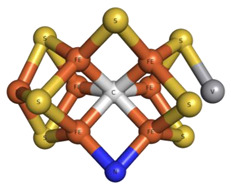	6FEA [87], 7ADR [88], 7ADY [88], 7AIZ [89]
8P8	FeV-Center VCFe_7_S_8_ 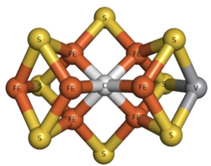	5N6Y [90]

**Table 2:** Single crystal X-ray structures of polyoxidovanadates-containing proteins available in the PDB since 2015. The identifier and the name/chemical structure of each V compound, as well as the respective PDB codes, are provided. ^a^ The publication associated with the PDB entry is not available.

V Species Identifier	V Species Name/Chemical Structure	PDB Codes
DVG	Divanadate Glycerol ester (DGV) V_2_C_3_H_10_O_8_ 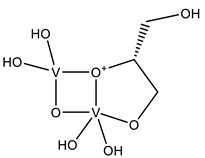	4ZI4 [58], 4ZN5 [58]
V4O	Cyclo-tetrametavanadate V_4_O_12_ 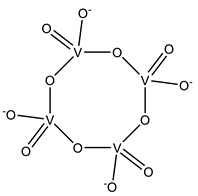	7ZU6 ^a^
VV6	Hexavanadate V_6_H_12_O_17_ 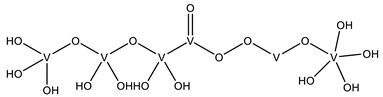	6HWR [81]
H1T	Heptavanadate V_7_O_20_ 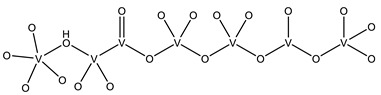	6HWR [81]

**Table 3:** Single crystal X-ray structures of V-nucleoside and V-nucleotide protein structures available in the PDB since 2015. The identifier and the name/chemical structure of each V compound, as well as the respective PDB codes, are provided. ^a^ The publication associated with the PDB entry is not available.

V Species Identifier	V Species Name/Chemical Structure	PDB Codes
KL2	Adenosine-2′,3′-vanadate VC_10_H_14_N_5_O_7_ 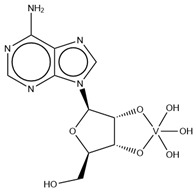	6RVZ [114]
H1Q	Adenosine divanadate V_2_C_10_H_13_N_5_O_11_ 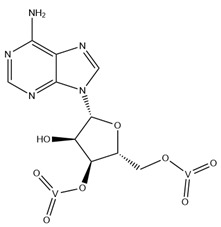	6HWR [81]
UVC	Uridine-2’,3’-vanadate VC_9_H_12_N_2_O_9_ 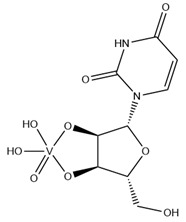	6YO1 [79], 7K1L [115]
AOV	ADP-vanadate VC_10_H_17_N_5_O_14_P_2_ 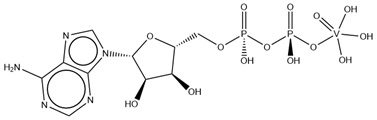	5I4E [116]
AD9	ADP Metavanadate VC_10_H_16_N_5_O_13_P_2_ 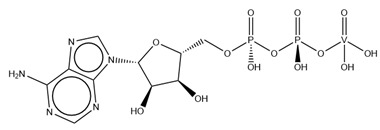	6PY9 [117], 6Z2S ^a^, 7B19 [118], 7B1A [118]
CVC	Cytidine-5′-monophosphate-2′,3′-vanadate VC_9_H_14_N_3_O_10_P 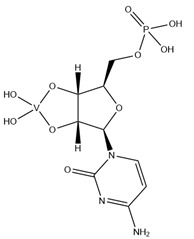	5EAO [119], 5EAQ [119]
Q61	Guanosine-5′-monophosphate-2′,3′-vanadate VC_10_H_12_N_5_O_10_P 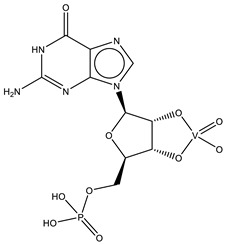	6UEY [120], 6UF1 [120]

**Table 4:** Structures of organic-based vanadium-complexes-containing proteins, obtained by single crystal X-ray diffraction analysis, available in the PDB since 2015.

Organic Molecule Name/Chemical Structure	PDB Codes
Picolinato 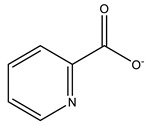	7P8R [132], 7Q0X [16]
Maltol 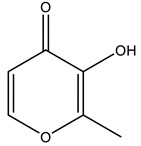	8AJ3 [75], 8AJ4 [75]
2,2′-bipyridine 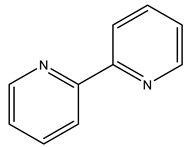	7Q0U [16]
1,10-phenanthroline 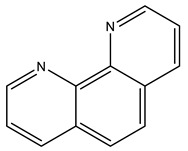	7Q0V [16], 7Q0W [16]
1-methyl-2-ethyl-3-hydroxy-4(1H)-pyridinone 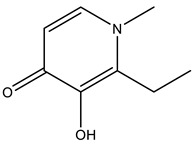	8OM8 [133], 8OMS [133], 8OMT [133]
8-hydroxyquinolinato 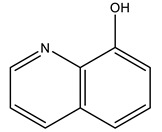	7QWH [134]

**Table 5:** Cryo-EM structures of V-containing proteins available in the PDB since 2015. The identifier and name/chemical structure of each vanadium compound, as well as the respective PDB codes, are provided.

V Species Identifier	V Species Name/Chemical Structure	PDB Codes
VO4	Orthovanadate VO_4_^3−^ 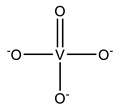	6VQT [167], 7BCW [168], 7D0A [169], 7MSM [170], 7MSZ [170], 7N5A [171], 7N5B [171], 7OU0 [172], 7ZNU [173], 7ZO9 [173], 7ZRD [174]
DVT	Decavanadate V_10_O_28_ 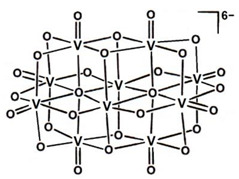	5WP6 [175]
AOV	ADP Orthovanadate VC_10_H_17_N_5_O_14_P_2_ 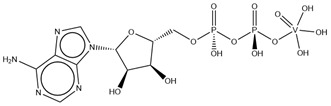	6MHZ [176], 6MI8 [176], 6RAJ [177], 6RAK [177], 7MDY [178], 7P06 [179], 8DMM [180]
AD9	ADP Metavanadate VC_10_H_16_N_5_O_13_P_2_ 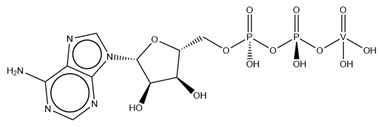	7CH8 [181]
